# Prevalence and outcomes of frailty in emergency laparotomy: a single-centre cohort study

**DOI:** 10.1007/s00068-026-03152-1

**Published:** 2026-03-23

**Authors:** Paul M. Rival, Cody Bellgrove, Muhammad Usama Ejaz, Joel M. Van Weel, Matthew A. R. Stokes, Charles H. C. Pilgrim

**Affiliations:** 1 Department of General Surgery, Frankston Hospital, Peninsula Health, 2 Hastings Rd, Frankston, VIC 3199 Australia; 2 Department of Medicine, Peninsula Health, Frankston, Victoria Australia; 3 National Centre for Healthy Ageing, Monash University, Frankston, Victoria Australia; 4 Department of General Surgery, The Alfred, Alfred Health, Melbourne, VIC Australia; 5 School of Translational Medicine, Monash University, Victoria, Australia; 6 School of Public Health and Preventative Medicine, Monash University, Victoria, Australia

**Keywords:** Emergency laparotomy, Frailty, Clinical frailty scale, Emergency surgery, Postoperative outcomes, Risk stratification

## Abstract

**Purpose:**

Frailty is common among patients undergoing emergency laparotomy and is associated with adverse postoperative outcomes, yet routine frailty assessment remains inconsistently implemented despite international guideline recommendations. This study evaluates the prevalence of frailty using three rapid assessment tools and examines their associations with postoperative outcomes following emergency laparotomy.

**Methods:**

We conducted a single-centre retrospective cohort study of adults undergoing open emergency laparotomy over a 12-month period. Frailty was assessed retrospectively using the Clinical Frailty Scale (CFS ≥ 5), Emergency Surgery Frailty Index (EmSFI ≥ 7), and five-item Modified Frailty Index (mFI-5 ≥ 2). Primary outcomes were 30- and 90-day mortality. Secondary outcomes included postoperative complications, ICU admission, hospital length of stay, and discharge destination. Unadjusted analyses were descriptive. Multivariable logistic regression models were constructed with frailty measures specified as the primary exposures and adjusted for age, sex, operative indication, and anaesthetist-assigned ASA grade.

**Results:**

Among 102 patients (median age 67 years, IQR 54–79; 53% female), frailty prevalence was 26% by CFS, 24% by EmSFI, and 27% by mFI-5, with 37% meeting at least one frailty threshold. In unadjusted analyses, patients living with frailty experienced higher rates of postoperative complications, ICU admission, longer hospital stay, and reduced likelihood of independent discharge home. After adjustment, frailty thresholds were not independently associated with 30- or 90-day mortality, while mFI-5–defined frailty was independently associated with ICU admission.

**Conclusion:**

Frailty assessment in emergency laparotomy identifies patients at increased risk of postoperative morbidity, ICU utilisation, and discharge dependence but does not independently predict short-term mortality after adjustment for key clinical factors.

## Introduction

Frailty is a complex clinical state characterised by a loss of physiological reserve and accumulation of deficits across physical, cognitive and functional domains [1–3], affecting 21% of Australians aged ≥ 65 years [4]. It is a well-established predictor of poor postoperative outcomes, including increased risk of morbidity, mortality and loss of independence [1], as well as prolonged hospital stays, and higher ICU admission rates [2].

It is estimated that almost 20,000 people undergo an emergency laparotomy in Australia and New Zealand each year [5]. Emergency laparotomies are a high-risk surgical procedure, with an overall 30-day mortality of 6.8% [5]. The Australian and New Zealand Emergency Laparotomy Audit - Quality Improvement (ANZELA-QI) found in 2020 that within their patient cohort, 52.5% of emergency laparotomies were performed on patients aged ≥ 65 years [6]. Therefore, a significant portion of patients undergoing emergency laparotomies in Australia are likely to be frail, and their systematic and early identification in the preoperative period would potentially improve postoperative outcomes. The UK’s Centre for Perioperative Care 2021 “Guideline for Perioperative Care for People Living with Frailty Undergoing Elective and Emergency Surgery” recommends the use of a validated frailty assessment for all patients aged ≥ 65 years [1]. This recommendation is reiterated by the ANZELA-QI that identified “preoperative frailty assessment” as 1 of 8 key performance indicators [6]. The importance of these recommendations is underscored through their finding that, prior to September 2021, only 28% of patients aged ≥ 65 years underwent preoperative frailty assessment prior to having an emergency laparotomy [6]. Overall, Australian guidelines are yet to integrate this recommendation into routine clinical practice.

At Frankston Hospital, a major tertiary centre that manages approximately 63,000 emergency presentations and admits over 65,000 inpatients each year, frailty assessments are not routinely performed when evaluating patient suitability for emergency laparotomy [7] Therefore, there is an opportunity to optimise the care of patients living with frailty by introducing a systematic approach to their preoperative identification. We have identified 3 rapid frailty assessments that have demonstrated efficacy in the perioperative period. They fit the criteria of being both quick (completed in <5 minutes) [2], and easy to perform. The first of these is the Clinical Frailty Score (CFS), a scale that ranks patient frailty from 1 (very fit) to 9 (terminally ill) and has previously been validated for use in emergency laparotomies [8]. The second is the Emergency Surgery Frailty Index (EmSFI), which scores patients based on 14 variables including clinical and laboratory criteria that has been validated as a reliable predictor of mortality in patients undergoing emergency surgeries [9]. The third is the five-item Modified Frailty Index (mFI-5), derived from comorbidity data, that has also been widely applied in surgical outcomes research, although it is increasingly recognised as a comorbidity-weighted risk index rather than a direct measure of biological frailty [10, 11]. While these tools are practical and rapid, they capture overlapping but distinct constructs, and their relative contributions to peri-operative risk, particularly when considered alongside established anaesthetic risk measures, remain incompletely defined.

The American Society of Anesthesiologists (ASA) physical status classification remains one of the most widely used predictors of peri-operative mortality and critical care utilisation. In emergency surgery, ASA grade may capture acute physiological derangement and illness severity more directly than frailty tools derived from baseline characteristics alone [12, 13]. The extent to which frailty provides independent prognostic information beyond ASA grade in emergency laparotomy populations is therefore uncertain and has important implications for how frailty screening should be operationalised in practice.

We conducted a single-centre retrospective cohort study of patients undergoing emergency laparotomy to evaluate the prevalence of frailty using three rapid, chart-derivable tools (CFS, EmSFI, and mFI-5), examine their associations with postoperative outcomes, and explore how frailty assessment might be integrated into a pragmatic, evidence-aligned pre-operative pathway. Rather than validating frailty tools as stand-alone mortality predictors, this study aimed to clarify their clinical utility for anticipating morbidity, resource utilisation, and discharge complexity in the context of emergency surgery.

## Methods

### Study design

This was a single-centre retrospective cohort study conducted at Frankston Hospital, a tertiary referral hospital within Peninsula Health, Victoria, Australia. The study evaluated adult patients undergoing emergency laparotomy under the Acute General Surgery Unit (AGSU) over a 12-month period (February 2024 to February 2025).

### Patient eligibility and selection

Eligible patients were adults aged ≥ 18 years who underwent an open emergency laparotomy, defined as an emergency abdominal operation involving entry into the peritoneal cavity. Patients were identified using the hospital electronic medical record (EMR) and operating theatre databases, with operative reports reviewed to confirm eligibility.

Exclusion criteria were:

1. Procedures not involving entry into the peritoneal cavity
2. Laparoscopic procedures without conversion to open surgery
3. Incomplete medical records precluding frailty or outcome assessment

Only the index emergency laparotomy admission was included for patients with multiple presentations during the study period.

### Frailty assessment

Frailty status was assigned retrospectively using three rapid, chart-derivable tools:

1. **Clinical Frailty Scale (CFS)**, scored from 1 (very fit) to 9 (terminally ill), with frailty defined as CFS ≥ 5.
2. **Emergency Surgery Frailty Index (EmSFI)**, a 14-item index incorporating clinical, physiological, and laboratory variables, with frailty defined as a score ≥ 7.
3. **Five-item Modified Frailty Index (mFI-5)**, derived from comorbidity and functional status variables, with frailty defined as a score ≥ 2.

Frailty scores were derived from admission notes, nursing documentation, allied-health assessments, discharge summaries, and correspondence describing pre-morbid functional status. Scores were intended to reflect baseline frailty prior to the index admission, rather than physiological deterioration occurring after presentation.

Three investigators (PR, CB, MUE) independently applied uniform scoring rules. Ambiguous cases were resolved by consensus review. Frailty tools were analysed individually and as a frailty signal burden, defined as meeting thresholds on one, two, or all three tools. For primary analyses, patients were categorised as non-frail (no frailty thresholds met) or living with frailty (≥1 threshold met).

### Variable and data collection

Data were extracted using a standardised data collection template. Baseline variables included age, sex, pre-admission residence (home or residential aged care), baseline mobility status (independent, aided, or dependent), and key comorbidities. Comorbidities were identified from documented past medical history within admission notes, anaesthetic records, and discharge summaries, with medication lists used to corroborate diagnoses where documentation was unclear. Operative indication was categorised as obstruction or perforation/ischaemia/sepsis/other. The ASA grade was recorded as documented by the intra-operative anaesthetist.

### Outcomes

The primary outcomes were:

- 30-day mortality
- 90-day mortality

Mortality outcomes were determined from EMR documentation and linked hospital records.

The secondary outcomes were:

- Any postoperative complication (Clavien–Dindo grades I–V)
- Major postoperative complications (Clavien–Dindo ≥ IIIa)
- Postoperative ICU admission
- Hospital length of stay (LOS), measured in days from admission to discharge or in-hospital death
- Discharge destination, categorised as independent discharge home, discharge home with services (hospital at home, rehabilitation, nursing home), or in-hospital death

### Statistical analysis

Continuous and ordinal variables were summarised as medians with interquartile ranges (IQR) and categorical variables as counts and percentages. Descriptive, unadjusted comparisons were performed to characterise differences between frailty groups. For descriptive analyses, comparisons between non-frail and frail groups (≥1 frailty signal) were conducted using the Mann–Whitney U test for continuous or ordinal variables and Fisher’s exact test for categorical variables. All *p* values from descriptive analyses were unadjusted and intended to describe group differences rather than infer independent associations.

For adjusted analyses, multivariable logistic regression was used, as outcomes of interest were binary and this approach allows estimation of adjusted odds ratios while accounting for potential confounding. Frailty measures were specified as the primary exposure variables of interest. Models were constructed for key postoperative outcomes, including 30-day mortality, 90-day mortality, major complications (Clavien–Dindo ≥ IIIa), ICU admission, prolonged hospital LOS (>10 days), and independent discharge home. Covariates were selected based on established associations with peri-operative risk and included age, sex, operative indication, and ASA grade. Frailty variables (CFS ≥ 5, EmSFI ≥ 7, mFI-5 ≥ 2, or ≥2-tool concordance) were entered one at a time into separate models to avoid collinearity. Given low event counts for mortality outcomes, penalised (Firth) logistic regression was used for mortality models. Adjusted odds ratios (OR) with 95% confidence intervals (CI) are reported. Statistical significance was defined as *p* ≤ 0.05.

## Results

### Cohort characteristics

During the 12-month study period, 102 patients underwent open emergency laparotomy and met inclusion criteria. The median age was 67 years (IQR 54–79), and 54 patients (53%) were female. Overall, 64 patients (63%) were classified as non-frail, while 38 patients (37%) met at least one frailty threshold. Of those living with frailty, 13 (12%) were positive on one frailty tool, 9 (9%) on two tools, and 16 (16%) on all three tools (Table 1).

**Table 1:** Patient characteristics

Category	Total	Non-Frail	Frail (≥1 score)	Descriptive p value (Non-Frail vs Frail)	Frail (1 score)	Frail (2 scores)	Frail (3 scores)
n (%)	102 (100)	64 (100)	38 (100)	n/a	13 (100)	9 (100)	16 (100)
Age: median [IQR 1–3]	67 [54–79]	62 [50–71]	77 [66–85]	<0.01	67 [58–75]	82 [75–89]	83 [73–88]
Male	48 (47)	27 (42)	21 (55)	0.22	6 (46)	4 (44)	11 (69)
Female	54 (53)	37 (58)	17 (45)		7 (54)	5 (56)	5 (31)
ASA grade: median [IQR 1–3]	3 [3–4]	3 [3–3]	4 [3–4]	<0.01	3 [3–4]	3 [3–4]	4 [4–4]
Pre-admission residence							
Home	98 (96)	64 (100)	34 (89)	0.02	13 (100)	8 (89)	13 (81)
Residential aged care	4 (4)	0 (0)	4 (11)		0 (0)	1 (11)	3 (19)
Pre-operative mobility status							
Independent	79 (77)	64 (100)	15 (39)	<0.01 (Independent vs non-independent)	8 (62)	4 (44)	3 (19)
Aided (walking and mobility aids)	17 (17)	0 (0)	17 (45)		4 (31)	4 (44)	9 (56)
Dependent on activities of daily living (≥1 helpers)	6 (6)	0 (0)	7 (18)		1 (8)	1 (11)	4 (25)
Key co-morbidities							
Diabetes	20 (20)	5 (8)	15 (39)	<0.01	6 (46)	3 (33)	6 (38)
HTN	48 (47)	22 (34)	26 (68)	<0.01	6 (46)	6 (67)	14 (88)
IHD/CCF	25 (25)	7 (11)	12 (32)	0.02	5 (38)	2 (22)	2 (13)
COPD/Asthma	14 (14)	6 (9)	8 (21)	0.14	2 (15)	1 (11)	5 (31)
Operative indication							
Obstruction	57 (56)	36 (56)	21 (55)	1	8 (62)	5 (56)	8 (50)
Perforation/ischaemia/sepsis/other	45 (44)	28 (44)	17 (45)		5 (38)	4 (44)	8 (50)

HTN: Hypertension; IHD: Ischaemic Heart Disease; CCF: Congestive Cardiac Failure; COPD: Chronic Pulmonary Obstructive Disease. Frailty signal defined as meeting the threshold for ≥1 frailty tool (Clinical Frailty Scale ≥ 5, Emergency Surgery Frailty Index ≥ 7, or Modified Frailty Index-5 ≥ 2). ASA grade was assigned by the anaesthetic team. Comorbidities were identified from documented past medical history within admission notes, anaesthetic records, and discharge summaries, with medication lists used to corroborate diagnoses where required

Frailty prevalence increased with age and functional dependence. Median age rose from 62 years (IQR 50–71) in non-frail patients to 77 years (IQR 66–85) in patients living with frailty (*p* < 0.01). Baseline mobility differed markedly between groups: all non-frail patients were independently mobile, compared with 39% of frail patients, among whom 45% required walking aids and 18% were dependent for activities of daily living (*p* < 0.01). Patients living with frailty also had higher comorbidity burden, including diabetes, hypertension, and ischaemic heart disease or congestive cardiac failure (all *p* < 0.02).

Anaesthetist-assigned ASA grade increased in parallel with frailty status. The median ASA grade was 3 (IQR 3–3) in non-frail patients compared with 4 (IQR 3–4) in patients living with frailty (*p* < 0.01). Patients frail by all three tools had a median ASA grade of 4 (IQR 4–4). Operative indication did not differ significantly between frailty groups, with obstruction accounting for approximately half of cases in both cohorts (Table 1).

### Unadjusted postoperative outcomes

Overall 30-day mortality was 10% (10/102), and 90-day mortality was 12% (12/102). Thirty-day mortality was higher among patients living with frailty compared with non-frail patients (16% vs 6%), although this difference did not reach statistical significance (*p* = 0.17). When stratified by frailty burden, 30-day mortality increased from 8% in patients frail by one tool to 25% in those frail by all three tools (Table 2).

**Table 2:** Patient groups and post operative outcomes

Category	Total	Non-Frail	Frail (≥1 score)	Descriptive p value (Non-Frail vs Frail)	Frail (1 score)	Frail (2 scores)	Frail (3 scores)
**n (%)**	102 (100)	64 (100)	38 (100)	n/a	13 (100)	9 (100)	16 (100)
30-day mortality, n (%)	10 (10)	4 (6)	6 (16)	0.17	1 (8)	1 (11)	4 (25)
90-day mortality, n (%)	12 (12)	5 (8)	7 (18)	0.12	1 (8)	2 (22)	4 (25)
Complications (Clavien–Dindo I-V), n (%)	58 (57)	29 (45)	29 (76)	<0.01	9 (69)	6 (67)	14 (88)
Major complications (Clavien–Dindo ≥ IIIa), n (%)	24 (24)	12 (19)	12 (32)	0.15	2 (15)	3 (33)	7 (44)
Number of ICU admission, n (%)	44 (43)	20 (31)	23 (61)	<0.01	4 (31)	5 (56)	14 (88)
Hospital length of stay, days: median [IQR 1–3]	10 [6–15]	9 [6–13]	12 [6–22]	0.02	6 [6–20]	13 [8–16]	13 [10–23]
Discharged home independently, n (%)	75 (74)	54 (84)	21 (55)	<0.01 (Independent vs non-independent)	9 (69)	6 (67)	6 (38)
Discharged home with services, n (%**)**	27 (26)	10 (16)	17 (45)		4 (31)	3 (33)	10 (62)

*‘*Services’ refer to new or increased community supports compared with pre-admission status

Postoperative morbidity was common. Any complication (Clavien–Dindo grades I–V) occurred in 58 patients (57%), with a higher incidence among patients living with frailty than non-frail patients (76% vs 45%, *p* < 0.01). Major complications (Clavien–Dindo ≥ IIIa) occurred in 24 patients (24%), with numerically higher rates among frail patients, particularly those frail by multiple tools, although these differences were not statistically significant (*p* = 0.15).

ICU admission was required for 44 patients (43%). Patients living with frailty were significantly more likely to require ICU admission than non-frail patients (61% vs 31%, *p* < 0.01), with ICU utilisation increasing stepwise with frailty burden, reaching 88% in patients frail by all three tools.

Hospital length of stay was longer among patients living with frailty (median 12 days, IQR 6–22) compared with non-frail patients (median 9 days, IQR 6–13; *p* = 0.02). Discharge outcomes also differed substantially: independent discharge home occurred in 55% of frail patients compared with 84% of non-frail patients (*p* < 0.01), while discharge home with services was more frequent among frail patients (45% vs 16%) (Table 2).

### Adjusted analyses

- **Mortality**

In adjusted models including age, sex, operative indication, and ASA grade, frailty defined by CFS, EmSFI, mFI-5, or concordance across ≥ 2 tools was not independently associated with either 30-day or 90-day mortality (Table 3). Adjustment for ASA grade attenuated the unadjusted association between frailty and mortality. Each unit increase in ASA grade was associated with higher odds of 30-day mortality (OR 9.39, 95% CI 2.27–38.74; *p* < 0.01) and 90-day mortality (OR 4.48, 95% CI 1.50–13.36; *p* < 0.01). Age, sex, and operative indication were not independently associated with mortality in adjusted models.

- **Major complications**

**Table 3:** Multivariable logistic regression analyses of key postoperative outcomes

Variable	n	Odds Ratio	95% CI	P value
**30-day mortality**				
Frail according to CFS	6	0.97	0.16 - 5.98	0.97
Frail according to EmSFI	5	0.67	0.11 - 4.30	0.67
Frail according to mFI-5	4	0.27	0.04 - 1.98	0.20
Frail on ≥ 2 frailty tools	5	0.38	0.13 - 3.81	0.68
**90-day mortality**				
Frail according to CFS	6	1.21	0.25 - 5.92	0.87
Frail according to EmSFI	5	0.53	0.10 - 2.90	0.46
Frail according to mFI-5	5	0.38	0.07 - 2.11	0.27
Frail on ≥ 2 frailty tools	6	0.61	0.11 - 3.45	0.57
**Major complications (CD ≥ IIIa)**				
Frail according to CFS	11	1.96	0.58 - 6.60	0.28
Frail according to EmSFI	9	1.09	0.32 - 3.69	0.89
Frail according to mFI-5	9	0.97	0.27 - 3.51	0.97
Frail on ≥ 2 frailty tools	10	1.86	0.49 - 7.10	0.36
**ICU admission**				
Frail according to CFS	19	1.79	0.53 - 6.07	0.35
Frail according to EmSFI	18	2.55	0.76 - 8.61	0.13
Frail according to mFI-5	21	3.77	1.09 - 13.05	0.04
Frail on ≥ 2 frailty tools	20	3.52	0.91 - 13.68	0.07
**Prolonged length of stay in hospital (>10 days)**				
Frail according to CFS	20	1.98	0.61 - 6.40	0.25
Frail according to EmSFI	17	1.59	0.52 - 4.91	0.47
Frail according to mFI-5	20	1.13	0.36 - 3.54	0.84
Frail on ≥ 2 frailty tools	19	1.42	0.40 - 5.01	0.58
**Discharged independently home**				
Frail according to CFS	15	0.54	0.15 - 1.89	0.33
Frail according to EmSFI	15	1.06	0.30 - 3.71	0.90
Frail according to mFI-5	17	0.87	0.25 - 3.08	0.83
Frail on ≥ 2 frailty tools	15	1.51	0.37 - 6.10	0.57

Models adjusted for age, sex, operative indication, and ASA grade. Frailty variables were entered one at a time into separate models to avoid collinearity

For major postoperative complications (Clavien–Dindo ≥ IIIa), frailty thresholds defined by CFS, EmSFI, mFI-5, or concordance across ≥ 2 tools were not independently associated with risk after adjustment (Table 3). Higher ASA grade remained independently associated with major complications (OR 2.32, 95% CI 1.07–5.01; *p* < 0.03), while female sex was associated with lower odds of major complications (OR 0.28, 95% CI 0.10–0.80; *p* < 0.02).

- **ICU admission**

In adjusted analyses, mFI-5–defined frailty was independently associated with ICU admission (OR 3.77, 95% CI 1.09–13.05; *p* < 0.04), whereas CFS- and EmSFI-defined frailty were not (Table 3). Concordance across ≥ 2 frailty tools demonstrated a trend toward increased ICU admission but did not reach statistical significance. Higher ASA grade was also independently associated with ICU admission (OR 4.02, 95% CI 1.72–9.40; *p* < 0.01).

- **Length of stay and discharge destination**

Prolonged hospital length of stay (>10 days) was independently associated with increasing age (OR 1.05 per year, 95% CI 1.01–1.08; *p* < 0.01) and female sex (OR 0.25, 95% CI 0.10–0.62; *p* < 0.01). Neither ASA grade nor frailty thresholds were independently associated with prolonged length of stay after adjustment.

Independent discharge home was less likely with increasing age (OR 0.94 per year, 95% CI 0.90–0.99; *p* < 0.02) and higher ASA grade (OR 0.29, 95% CI 0.12–0.70; *p* < 0.01). Frailty thresholds were not independently associated with discharge destination after adjustment (Table 3).

## Discussion

### Principal findings

In this single-centre cohort of patients undergoing emergency laparotomy, frailty was prevalent and associated with substantially worse unadjusted postoperative outcomes, including higher complication rates, greater ICU utilisation, longer hospital length of stay, and reduced likelihood of independent discharge. However, after adjustment for age, sex, operative indication, and ASA grade, frailty thresholds derived from three rapid tools (CFS, EmSFI, and mFI-5) were not independently associated with 30- or 90-day mortality. These findings suggest that frailty and ASA capture related but distinct dimensions of peri-operative risk in emergency laparotomy, with frailty more closely aligned to postoperative morbidity and recovery complexity than short-term mortality.

### Frailty and postoperative morbidity: consistency with existing literature

The observed association between frailty and postoperative morbidity is consistent with peri-operative frailty literature demonstrating that frailty identifies patients at heightened risk of complications, prolonged hospitalisation, and loss of functional independence, especially following emergency laparotomy. Isand et al. recently demonstrated that patients with frailty undergoing emergency laparotomy experienced a 1.38-fold longer delay to surgery and a 1.24-fold longer postoperative LOS, even after adjusting for indication, sepsis, intraperitoneal soiling, malignancy status, and delay to surgery [14]. In addition, in a narrative review of older adults undergoing elective surgery, Subramaniam et al. highlighted frailty as a strong predictor of adverse postoperative outcomes and argued that frailty screening should act as a practical trigger for enhanced multidisciplinary, person-centred peri-operative management, rather than functioning solely as a risk label [15]. They noted that the interventional evidence base remains limited, but that comprehensive geriatric assessment (CGA) and optimisation has been associated with fewer complications and shorter LOS, and may be most efficiently targeted to patients identified as frail to direct finite geriatric and allied-health resources where benefit is most likely. Although this review focuses on elective surgery, its core implication aligns with our emergency laparotomy findings: frailty is most clinically actionable when used to prompt early multidisciplinary input, mobilisation, delirium prevention, nutrition support, medication review, and anticipatory discharge planning, aimed at mitigating morbidity and supporting recovery, rather than being used as a stand-alone mortality prediction tool.

Importantly, our data also demonstrate a graded relationship between frailty burden and postoperative morbidity, with progressively higher ICU utilisation and reduced independent discharge as the number of positive frailty tools increased. This burden-based signal aligns with the Emergency Laparotomy and Frailty (ELF) study, in which increasing CFS categories were associated with stepwise increases in postoperative complications and institutional discharge, even after accounting for age and comorbidity [8].

### Mortality prediction: why ASA outperforms frailty in emergency laparotomy

A key finding of this study is that frailty thresholds did not independently predict short-term mortality once adjustment was made for key clinical covariates, including ASA grade. This contrasts with some elective surgery cohorts, where frailty has demonstrated independent associations with mortality, but aligns closely with contemporary peri-operative anaesthesia literature focused on emergency and high-acuity surgery. McIsaac et al. demonstrated that while frailty is strongly associated with death or new disability, acute physiological illness severity substantially attenuates its independent association with mortality, particularly in urgent and emergent procedures [16]. Another study similarly suggests that peri-operative outcomes in emergency surgery are best explained by the interaction between baseline vulnerability and acute physiological stress, rather than by frailty alone [17].

In emergency laparotomy, where sepsis, haemodynamic instability, metabolic derangement, and organ dysfunction are common, ASA grade may better reflect acute physiological burden at the time of surgery than frailty tools derived primarily from baseline characteristics. In our cohort, inclusion of ASA grade in adjusted models markedly attenuated the association between frailty and mortality. This finding mirrors those of Hackett et al., who reported that ASA grade demonstrated stronger discrimination for mortality than frailty indices in emergency laparotomy, particularly when multiple covariates were included in adjusted models [12]. This does not diminish the clinical relevance of frailty assessment, but rather underscores that frailty and anaesthetic risk stratification address complementary aspects of peri-operative risk.

### Interpreting differences between frailty tools

The absence of an independent mortality association across all three frailty tools in adjusted analyses highlights that these instruments assess overlapping but distinct constructs. The Clinical Frailty Scale primarily reflects baseline functional reserve and dependence; the Emergency Surgery Frailty Index incorporates acute clinical and laboratory parameters; and the mFI-5 functions predominantly as a comorbidity-weighted risk index.

Notably, mFI-5 defined frailty retained an independent association with ICU admission in our cohort. This finding aligns with the construction of the mFI-5, which is derived from comorbidity and functional dependence variables and is heavily weighted toward cardiopulmonary disease, diabetes, and chronic illness burden. In validation studies using American College of Surgeons NSQIP data, the mFI-5 demonstrated strong correlation with the original 11-item index and comparable discrimination for postoperative complications and mortality, supporting its role as a pragmatic risk index rather than a direct measure of biological frailty [11]. In emergency laparotomy, such comorbidity-weighted risk may reasonably influence peri-operative escalation decisions, including invasive monitoring and postoperative ICU admission, without necessarily translating into excess short-term mortality once acute illness severity is accounted for. Therefore, mFI-5 based indices may not be directly interchangeable with clinical frailty constructs, instead positioning them as markers of comorbidity-related peri-operative risk [11]. Our findings support this distinction and caution against interpreting mFI-based indices as interchangeable with clinical frailty constructs.

### Clinical implications: reframing the role of frailty in emergency laparotomy

**Fig. 1:**
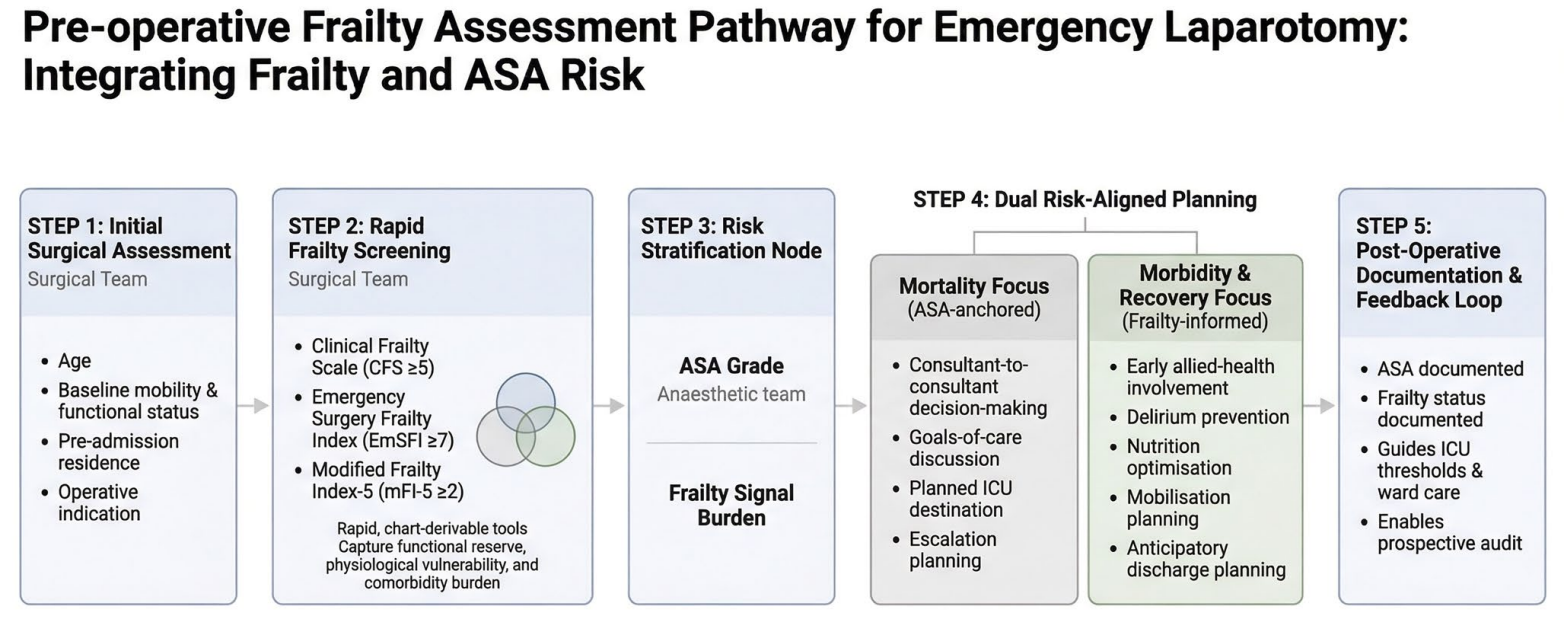
Conceptual pre-operative frailty assessment pathway for emergency laparotomy. Rapid frailty screening using the Clinical Frailty Scale (CFS), Emergency Surgery Frailty Index (EmSFI), and five-item Modified Frailty Index (mFI-5) is integrated with American Society of Anesthesiologists (ASA) grade–based physiological risk stratification. ASA grade anchors mortality-focused escalation planning, while frailty signals inform morbidity- and recovery-oriented multidisciplinary optimisation and discharge planning

Taken together, these findings suggest that frailty assessment in emergency laparotomy should be viewed as a complementary component of peri-operative risk stratification rather than a stand-alone mortality prediction tool. On the basis of our results, we propose a conceptual pre-operative frailty assessment pathway (Fig. 1) that integrates rapid frailty screening with ASA-based physiological risk assessment.

In this model, ASA grade anchors mortality-focused escalation planning, while frailty signals inform morbidity- and recovery-oriented optimisation, including early multidisciplinary input and anticipatory discharge planning. This proposed pathway is hypothesis-generating and would require prospective evaluation. Future pilot studies should assess the feasibility, uptake, and clinical impact of embedding structured frailty screening into emergency laparotomy workflows.

### Strengths and limitations

This study adds to the literature by directly comparing three rapid frailty tools within a contemporary emergency laparotomy cohort and examining their associations with postoperative outcomes in adjusted analyses. The use of pragmatic, chart-derivable tools reflects real-world practice and aligns with current quality-improvement priorities, particularly in time-critical emergency surgical settings where prospective bedside assessment may not be feasible.

Several limitations warrant consideration. First, the retrospective design and modest sample size limit statistical power, particularly for mortality outcomes, and increase the risk of type II error. Second, frailty was assessed retrospectively using documentation-derived data rather than prospective bedside evaluation. Although prior studies have demonstrated reasonable agreement between retrospective and prospective frailty scoring, particularly for the Clinical Frailty Scale, incomplete or variable documentation may have led to misclassification, likely biasing associations toward the null [18, 19]. Third, while frailty tools are most commonly validated in populations aged ≥ 65 years, younger adults were included to reflect real-world emergency laparotomy practice; interpretation of frailty measures in patients aged < 65 years should therefore be cautious [20]. As a single-centre study, findings may not be fully generalisable to institutions with different patient demographics, peri-operative pathways, or access to multidisciplinary support. Finally, outcomes were limited to 90-day follow-up due to the absence of registry linkage or longitudinal mortality data, and longer-term functional and survival outcomes could not be assessed.

## Conclusion

In this emergency laparotomy cohort, frailty was common and associated with postoperative morbidity, ICU utilisation, and discharge complexity on unadjusted analysis, but was not independently associated with short-term mortality after adjustment. Mortality appeared more closely aligned with acute physiological risk reflected by ASA grade, whereas frailty captured vulnerability to complications and recovery burden. These findings support viewing frailty and anaesthetic risk stratification as complementary domains of peri-operative assessment. Prospective studies should evaluate whether integrating structured frailty screening into emergency laparotomy workflows improves patient-centred outcomes.

## Data Availability

Variable and data collection (Methods) Data were extracted using a standardised data collection template. Baseline variables included age, sex, pre-admission residence (home or residential aged care), baseline mobility status (independent, aided, or dependent), and key comorbidities. Comorbidities were identified from documented past medical history within admission notes, anaesthetic records, and discharge summaries, with medication lists used to corroborate diagnoses where documentation was unclear. Operative indication was categorised as obstruction or perforation/ischaemia/sepsis/other. The ASA grade was recorded as documented by the intra-operative anaesthetist.
